# Obstacles to Enforcing Regulations Requiring the Tuberculin Test in Inter-State Cattle Traffic1Read at the Thirty-seventh Annual Meeting of the American Veterinary Medical Association, Detroit, September, 1900.

**Published:** 1900-11

**Authors:** Austin Peters

**Affiliations:** Chairman of the Massachusetts Cattle Commission


### OBSTACLES TO ENFORCING REGULATIONS REQUIRING THE TUBERCULIN TEST IN INTER-STATE CATTLE TRAFFIC.[1]

By Austin Peters, M.R.C.V.S.,
CHAIRMAN OF THE MASSACHUSETTS CATTLE COMMISSION.

(Concluded from page 597.)

The Massachusetts Cattle Commission has been impeded and imposed upon in every possible way that many of the drovers could devise. Most of the dealers undoubtedly thought when these regulations were first adopted, five years ago, that tuberculosis was a fad and a temporary matter; that it was of little importance, and that tuberculin did not amount to anything. A farmer in one of the northern New England States does not like to sell a cow subject to the test and have her left on his hands if she reacts; the drover does not like to buy a cow out and out and have her react, because he has to sell her at a loss near home, her value being diminished if she turns out to be an animal he is not allowed to bring into Massachusetts. The result has been that a number of the dealers have done their best to corrupt the veterinarians or alleged veterinarians making the tests, and induce them to make out certificates without using tuberculin at all, and in many instances have succeeded in doing so. When the Massachusetts Cattle Commission finds that a man is doing dishonest work it refuses to accept his tests, and the drover then has to find a new

[1] Read at the Thirty-seventh Annual Meeting of the American Veterinary Medical Association, Detroit, September, 1900.

man, and, if possible, corrupt him. There have been a few exceptions to this rule, when the culprit has acknowledged that he has done wrong, and has promised to turn over a new leaf when the disgrace of his dishonesty has been pointed out to him, and he has been reinstated.

In localities where an occasional carload of cows is shipped into Massachusetts I think that the testing has been in the main properly done; but where the cattle are shipped every week, as they are from certain points in Maine, New Hampshire, and Vermont, corrupt methods have developed. Two years ago last spring the Massachusetts Cattle Commission had a list printed of men whose tests it would accept, after dropping a number of names from the old list, and it is now time to prepare a new one; the chief reason for delay is the fact that just when it seems that the names of only reliable men are ready it is found that another good man has gone wrong.

Another reason for dishonest work, in some instances, is due to competition among the veterinarians, who cut prices in order to obtain a certain drover's patronage until they reduce the price to such a rate that a man cannot afford to test the cattle and use tuberculin, and so make out the papers without the formality of a test. This has been a very foolish cause for this kind of work, as there are so few men on the list now that they could all agree to a good price and obtain it.

Occasionally a tuberculous cow may be honestly tested and fail to react—that is, she may be tested by a man one week and refused a certificate, and then the owner may have another veterinarian test her the following week without informing him that she has reacted once, and thus obtain a certificate of health because she fails to react when tested the second time; or a drover may have a cow of which he is suspicious, and inject her with a heavy dose of tuberculin himself, and when she recovers have her tested by the veterinarian. Occasionally a badly diseased cow may fail to react, but these cases ought to be perceptible from the physical condition of the creature; but when a man is testing a large number, and has gotten into the habit of depending entirely on tuberculin, he may overlook such a case. In my experience, a cow's failing to react to a second test made soon after the first one is not as frequent as many persons believe; in the majority of cases an animal that has given a marked reaction once is very likely to react again.

Numerous specific instances of dishonest work might be given. Last autumn an Ontario graduate, supposed to be one of the leading veterinarians of New Hampshire, was called to Dracut, Mass., to test a cow just brought in from across the line, held in quarantine until a certificate of test was sent in. Soon after, suspecting that all was not right, I proceeded to Dracut, and went with the inspector of animals to see about releasing the cow. I asked the owner if she had been tested. He said: "Oh, yes; the man came and stuck the tubule right into her; took it out of his pocket and stuck it in." Asked how long he was there and how many times he called, he said: "He only seen him once, and he was only there a few minutes." All he had done was to take the cow's temperature, make a physical examination, and then give a certificate of tuberculin test. The cow failed to pass when tested properly later. This veterinarian called to see me and denied he ever did such a thing before, but acknowledged his transgression in the case I caught him on, and said he would be very careful in the future. The words were hardly cold from his mouth before he was called upon to test a lot of cows to be sold at auction in Southern New Hampshire, some of which might be brought into Massachusetts. A number were brought in with his certificate and held by the Commission and tested; several reacted, showing that they either were not tested properly or probably not at all. It is needless to say that his tests will not be accepted any longer by our board. This is only one example of a number that I might give.

Early in June a large Jersey breeder in Pennsylvania had Dr. Francis Bridge test a number of cattle he intended selling at auction, and sold them with his certificates. A neighbor was going to have an auction of Jersey cattle at about the same time, and he thought it would be a favorable opportunity to have Dr. Bridge test his. I believe there were quite a large number—over one hundred, if I am correctly informed—and some twenty odd failed to pass, and Dr. Bridge refused to give certificates. The owner had a local veterinarian test the cattle, who gave certificates on some, if not all, and they were sold at the auction with the other man's certificate. At the sale the statement was given out that Dr. Bridge did not test all the animals, as quite a little bunch was overlooked until after he had gone, and, therefore, they had been tested by another doctor. Several cattle from this sale were brought into Massachusetts, but all had been tested by Dr. Bridge. If any tested by the other man had been shipped into the State they would have been held and retested by the Cattle Commission, with, I believe, interesting results.

The Bureau of Animal Industry is in the best position to obtain honest tuberculin tests, as it holds the cattle in quarantine at the port of entry, and has its own agents to test them, and therefore knows the work is honestly done.

The greatest obstacle to the enforcement of laws or regulations requiring a tuberculin test in the inter-state cattle traffic is dishonesty.

First, there are the avarice and lack of honesty among some cattle dealers and drovers, which lead them to object to the test because it interferes with their profits.

Secondly, the dishonesty of certain veterinarians, who disgrace and dishonor a profession which should be a useful and honorable one by claiming to be members of it.

Possibly there is more excuse for the cattlemen, as many of them think tuberculin is a humbug; that the test is of no value, and that these regulations are a passing fashion—not come to stay. I do not wish it to be understood that I regard all our cattle dealers and drovers dishonest or dishonorable, as there are a number of men among them of the strictest integrity and reliability, but it is greatly to be deplored that many of them are not.

The veterinarians ought to know better than to do dishonest work, and should be glad to coöperate with the authorities in any State in diminishing a scourge to the farmer, even though too many farmers are so ignorant and short-sighted as to fail to appreciate what is being done for them. As to the danger to the public health, I think that is a matter that has been overestimated. The attempt to terrorize the community with the dangers of the use of dairy products, on account of tuberculosis, by certain veterinarians whom the people have suspected of wanting salaries has done much to cause a reaction against the work and lead to a lack of confidence in the profession, such as is so well exemplified in the very wise legislation already alluded to in the State of Connecticut. Much of the trouble seems to be due to a lack of honesty among certain dishonorable members of the profession. What other remedy there is except refusing to accept their tests I do not know. They ought certainly to be expelled from any veterinary associations to which they belong, although most of the offenders belong to a class that do not join associations. Dealers and drovers or breeders who sell cattle with fake tests ought to be prosecuted for obtaining money under false pretences, and a breeder who will do such a thing ought to be expelled from any breeders' association, and his cattle ought to be refused registry in the herd-book.

A lack of honesty seems to be a national failing. Parents should bring up their boys to realize that it is a sin and a disgrace to steal, and that "a lie is an abomination to the Lord." Our veterinary schools should lay greater stress on professional integrity than at present; and if some means could be devised for disciplining the rascals, even to revoking their diplomas, if that is possible, it would be a benefit. "Honesty is the best policy;" but my experience with men has been that a man who is not honest as a matter of principle is not very likely to be so as a matter of policy.

Other obstacles to the enforcement of regulations requiring the tuberculin test may be carelessness on the part of railroad companies in seeing that a shipper to a point outside a quarantine station has a permit. It occasionally happens that a freight agent may accept a shipment of cattle from a man who has not secured a permit without notifying the authorities in the State to which the cattle are shipped. This can be remedied by reporting the local freight agent to the general freight agent of the road whenever such an instance is heard of, and in time the work will be so perfected as to have no such infringement of the rules, as they are broken more from not understanding them than from any direct intention to disregard the law.

Another obstacle that will always exist on a small scale is the trading back and forth of cattle by farmers in adjoining towns located in different States; but the number of animals exchanged in this way is limited. The necessary rules or laws may be enforced here to a certain extent, but there will always be a number of instances where they will be quietly disregarded.

I have necessarily confined myself chiefly to the condition of affairs in New England, and more especially to Massachusetts, as this is where my personal experience lies; but what I have said will probably apply to a certain extent to other sections, and it may be that the trials we have been called upon to endure may result in making it easier for others later.

